# NOX4 blockade suppresses titanium nanoparticle-induced bone destruction via activation of the Nrf2 signaling pathway

**DOI:** 10.1186/s12951-022-01413-w

**Published:** 2022-05-23

**Authors:** Wei Wang, Xiaolong Liang, Xin Liu, Jiaxiang Bai, Wei Zhang, Wenming Li, Tianhao Wang, Meng Li, Zerui Wu, Liang Chen, Huilin Yang, Ye Gu, Yunxia Tao, Jun Zhou, Huaiyu Wang, Dechun Geng

**Affiliations:** 1 Department of Orthopedics, The First Affiliated Hospital of Soochow University, Suzhou, 215006 Jiangsu China; 2 Department of Orthopedic Surgery, The First Affiliated Hospital of University of Science and Technology of China, Hefei, 230001 Anhui China; 3 Department of Orthopedic Surgery, The Affiliated Hospital of Xuzhou Medical University, Xuzhou, 221002 Jiangsu China; 4 Department of Orthopedics, Changshu Hospital Affiliated to Soochow University, First People’s Hospital of Changshu City, Changshu, China; 5 Center for Human Tissues and Organs Degeneration, Shenzhen Institute of Advanced Technology, Chinese Academy of Sciences, Shenzhen, 518055 China

**Keywords:** Periprosthetic osteolysis, Titanium nanoparticles, Osteoclastogenesis, NOX4, Nrf2

## Abstract

Periprosthetic osteolysis (PPO) triggered by wear particles is the most severe complication of total joint replacement (TJR) surgeries, representing the major cause of implant failure, which is public health concern worldwide. Previous studies have confirmed the specialized role of osteoclast-induced progressive bone destruction in the progression of PPO. Additionally, the reactive oxygen species (ROS) induced by wear particles can promote excessive osteoclastogenesis and bone resorption. Nicotinamide adenine dinucleotide phosphate oxidase 4 (NOX4), a cellular enzyme, is considered to be responsible for the production of ROS and the formation of mature osteoclasts. However, NOX4 involvement in PPO has not yet been elucidated. Therefore, we investigated the mechanism by which NOX4 regulates osteoclast differentiation and the therapeutic effects on titanium nanoparticle-induced bone destruction. We found that NOX4 blockade suppressed osteoclastogenesis and enhanced the scavenging of intracellular ROS. Our rescue experiment revealed that nuclear factor-erythroid 2-related factor 2 (Nrf2) silencing reversed the effects of NOX4 blockade on ROS production and osteoclast differentiation. In addition, we found increased expression levels of NOX4 in PPO tissues, while NOX4 inhibition in vivo exerted protective effects on titanium nanoparticle-induced osteolysis through antiosteoclastic and antioxidant effects. Collectively, these findings suggested that NOX4 blockade suppresses titanium nanoparticle-induced bone destruction via activation of the Nrf2 signaling pathway and that NOX4 blockade may be an attractive therapeutic approach for preventing PPO.

**Graphical Abstract:**

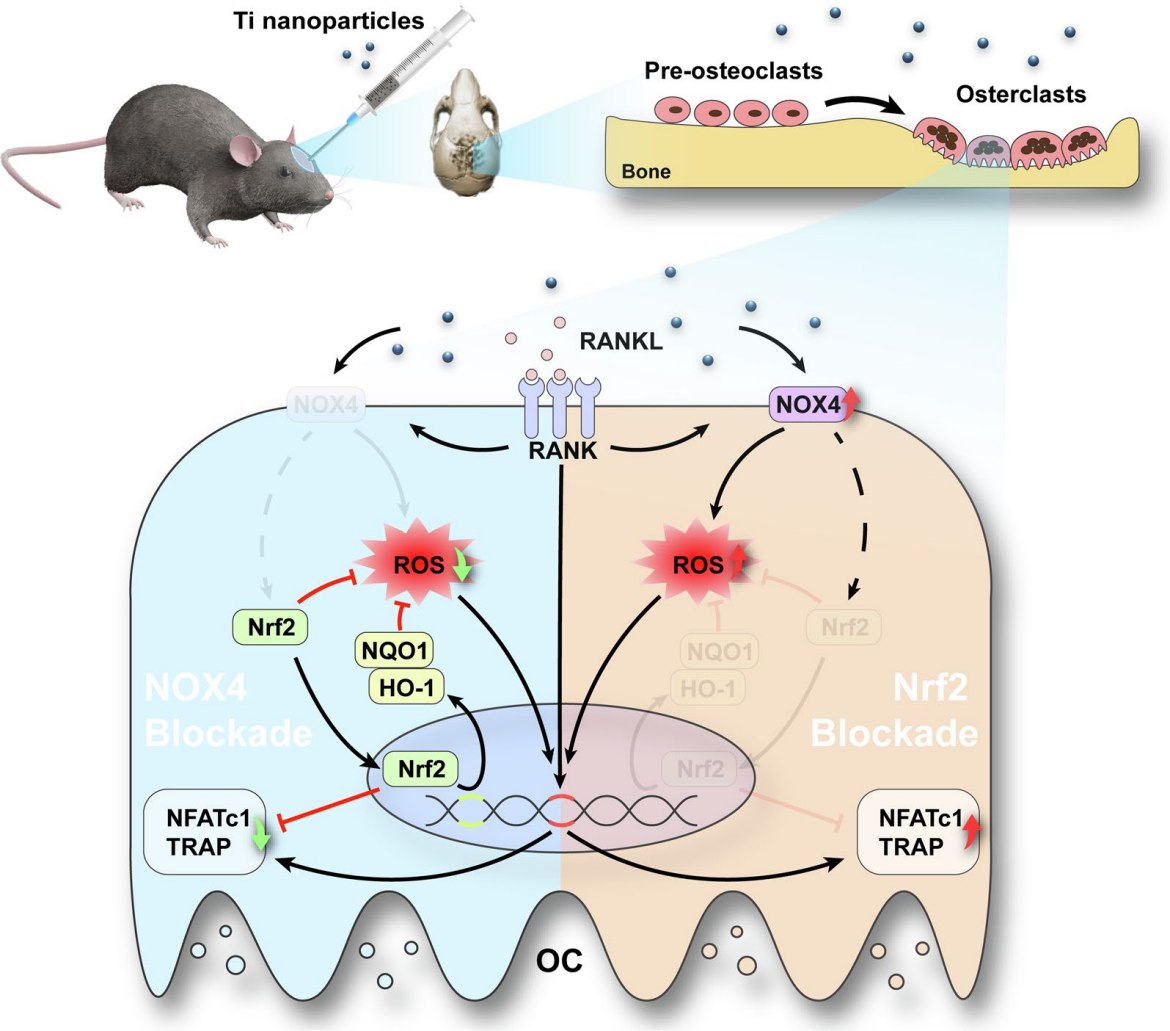

**Supplementary Information:**

The online version contains supplementary material available at 10.1186/s12951-022-01413-w.

## Introduction

Total joint replacement (TJR) is an essential surgical strategy for end-stage arthritis. Due to the increase in the aging population, the number of patients undergoing TJA is rapidly rising. In the USA, the total annual count of total hip arthroplasties (THAs) by 2040 is predicted to be 1,429,000 and that of primary total knee arthroplasties (TKAs) is predicted to be 3,416,000 by 2040. The percent increases in primary THA and primary TKA compared to the 2014 numbers were 284% and 401%, respectively [1]. However, periprosthetic osteolysis (PPO) and aseptic loosening are the most common complications of TJR surgeries and are almost unavoidable issues that lead to a reduced quality of life and increased risk of instability in these patients [2, 3]. In addition, the mechanism of PPO has not been fully elucidated, which increases the difficulty in the treatment of PPO.

The progressive bone destruction induced by wear particles is an important characteristic of PPO [2, 4]. Friction during movement of artificial joints induces the production of implant-derived nanoparticles, including titanium (Ti), chromium and polyethylene, which can stimulate multiple cells, such as macrophages and lymphocytes, to secrete different kinds of proinflammatory chemokines and cytokines. These molecules in turn recruit osteoclast precursors and act on osteoblasts and other target cells to promote the secretion of receptor activator of nuclear factor-κB ligand (RANKL). After RANKL stimulation, osteoclast precursors mutually fuse to form excess multinucleated mature osteoclasts on the surface of peri-implants, eventually resulting in extensive bone destruction [2, 4–6]. Osteoclasts play specialized roles in the pathologies of PPO, but the related mechanism remains unclear [7, 8]. Therefore, it is desirable to elucidate the mechanisms of PPO, reveal its pathogenesis and discover novel therapeutic targets and efficacious drugs for treatment of this condition.

Osteoclastogenesis is coupled with increased levels of reactive oxygen species (ROS), which regulate bone homeostasis through two mechanisms. Under physiological conditions, ROS are beneficial and essential as intracellular signaling agents and for bone homeostasis [9, 10]; however, relatively high amounts of ROS promote osteoclastogenesis to further enhance osteoclast bone resorption [10, 11]. Previous studies have suggested that hyperactive osteoclasts and increased oxidative stress level-induced bone resorption are predominant in the initiation of PPO. Further evidence has unanimously indicated that the inhibition of ROS through various mechanisms can restrain osteoclast differentiation and bone resorption [10–13]. Therefore, regulating unbalanced ROS production could be a strategy to treat PPO. Nicotinamide adenine dinucleotide phosphate oxidase 4 (NOX4), one of the members of the nicotinamide adenine dinucleotide phosphate oxidase family, is induced during differentiation in many cells. Accumulating evidence indicates that NOX4 is involved in the production of ROS [11, 14–16]. More importantly, NOX4 is constitutively active and produces H_2_O_2_ without cytosolic activator proteins, while H_2_O_2_ has been confirmed to contribute to the development of aseptic loosening [17, 18]. Studies have shown that osteoclastogenesis and the expression of recycling markers of bone resorption are decreased in mice with NOX4 knockout [19, 20]. Nevertheless, the relevant mechanism of NOX4 in the differentiation and maturation of osteoclasts is still unknown [11], and whether NOX4 is involved in PPO by enhancing osteoclastic bone resorption and ROS production has not yet been reported.

In the current study, we report the function of NOX4 in wear particle-mediated PPO and explored possible mechanisms using RANKL-induced osteoclastogenesis in vitro and a Ti nanoparticle-induced model of osteolysis in vivo. We revealed that NOX4 blockade regulates bone resorption by preventing hyperactivity of osteoclasts. The underlying mechanism relies on enhanced ROS scavenging and the activation of nuclear factor-erythroid 2-related factor 2 (Nrf2) and its downstream signaling pathway. Additionally, NOX4 blockade suppresses Ti nanoparticle-induced bone destruction through anti-osteoclastic and antioxidant activities. Collectively, these findings indicated that NOX4 blockade represents an attractive therapeutic approach to prevent PPO.

## Methods and materials

### Drugs and reagents

Ti nanoparticles were obtained from Nanjing Emperor Nano Materials Company. RNA interference sequences were purchased from GenePharma (Suzhou, China). Murine RANKL was purchased from R&D Systems (Minneapolis, USA). GKT137831 was purchased from ApexBio (Boston, USA). Dulbecco’s modified Eagle’s medium (DMEM/high glucose) was purchased from VivaCell (Shanghai, China) and fetal bovine serum (FBS) was obtained from HyClone (Logan, USA). CCK-8 assay kits were obtained from ApexBio (Boston, USA). The primary antibodies used in our study included NFATc1 (A1539, ABclonal, Wuhan, China), MMP-9 (A0289, ABclonal), NOX4 (A11274, ABclonal), HO-1 (ab189491, Abcam Cambridge, UK), SOD2 (ab137037, Abcam), and Nrf2 (A0674, ABclonal). Secondary antibody was purchased from Multisciences (Hangzhou, China).

### Cell culture

RAW264.7 cells were used as osteoclast precursors in this study. The cell line was obtained from the Chinese Academy of Sciences (Shanghai, China) and cultured on high-glucose media consisting of 10% FBS and penicillin/streptomycin antibiotics. The media were changed every 2–3 days.

### Osteoclast formation assays

RAW264.7 macrophages were seeded into 48-well plates (3 × 10^4^ cells per well). After overnight adherence, macrophages were cultured in media consisting of 50 ng/ml murine RANKL until the formation of mature osteoclasts. Then, TRAP staining was performed according to the manufacturer’s protocol (Bizhong Bio, Suzhou) after the cells were fixed with 4% paraformaldehyde. TRAP-positive osteoclasts (≥ 3 nuclei) were photographed via bright microscopy (Zeiss, Dresden, Germany) and quantified using ImageJ software (Bethesda, USA).

### F-actin staining

After the formation of mature osteoclasts, F-actin ring staining was performed. In brief, after fixation with 4% paraformaldehyde, the cells were stained with phalloidin (1:200, Yeasen, China), and then, the nuclei were stained with DAPI. Eventually, images were obtained under a fluorescence microscope (Zeiss, Germany) and quantified using ImageJ software.

### Cell transfection

RAW264.7 macrophages were transfected with siRNA and GP-transfect-Mate (GenePharma) according to the manufacturer’s protocol. In brief, the cells were reseeded in 12-well plates (2 × 10^5^ cells/well) and left overnight to adhere. Then, the cells were transfected with 20 nM siRNA, and the medium was changed to complete high-glucose medium overnight and continually maintained for 72 h. Finally, total proteins were harvested, and western blotting was applied to detect the expression of NOX4 and Nrf2.

### Cytotoxicity assessment

A CCK-8 assay was used to detect the cytotoxicity of GKT137831. Briefly, RAW 264.7 cells were seeded in 96-well plates and cultured and treated with GKT137831 at various concentrations after overnight adherence. The optical density (OD) at 450 nm was measured by a microplate reader (BioTek, USA) according to the spectrophotometric absorbance.

### Bone resorption assays

A bone resorption assay was employed to detect osteoclastic function. RAW264.7 macrophages were reseeded onto bovine bone slices (JoyTech Biotechnology, Zhejiang, China) and subsequently induced with RANKL (50 ng/ml). Ten days later, the cells were removed from the bovine bone slices using a sonicator. After modified gradient ethanol dehydration, the bone slices were completely dried by the critical-point drying method. Then, the bone slices were sputtered with gold in an airless spray unit. Resorption pits on the bone slices were observed using an FEI Quanta 250 scanning electron microscope (Hillsboro, USA) and quantified using ImageJ software (Bethesda, USA). The pit area was normalized to the whole area of the field of bovine bone slices.

### Quantitative real-time PCR

Total RNA was isolated by TRIzol reagent (Beyotime, Shanghai, China), and then, an equal amount of RNA was used for reverse transcription. MonAmp™ ChemoHS qPCR Mix (Monad, Suzhou, China) was employed to perform RT-PCR amplification via a CFX96™ thermal cycler (Bio-Rad Laboratories). Additional file 1: Table S1 lists the sequences of murine primers.

### Western blot assays

Total protein was extracted with RIPA lysis buffer. The same amount of protein was separated by SDS-PAGE and transferred to a nitrocellulose membrane. The membranes were successively incubated with primary antibodies and horseradish peroxidase-conjugated secondary antibodies. The relative gray level was detected via enhanced chemiluminescence (ECL, NCM, China) and then quantitated by ImageJ software (Bethesda, USA).

### ROS detection

ROS production was detected by a 2,7-dichlorodihydrofluorescein diacetate (DCFH-DA) staining kit (Beyotime, Shanghai, China). In brief, cells in the different groups were added to FBS-free media with DCFH-DA and subsequently incubated for 30 min. After removal of the media, the cells were fixed and observed under a fluorescence microscope and then quantitated by ImageJ software.

### Human tissue

Human joint synovial samples were obtained from primary TJAs of osteoarthritis patients or revision TJAs of patients with PPO in the First Affiliated Hospital of Soochow University, with three samples in each group. Synovial samples were closely associated with the response to implant wear debris and were collected from regions of bone resorption during revision or primary arthroplasty. Ethics approval was obtained by the Ethics Committee of the First Affiliated Hospital of Soochow University (2021 Ethics approval no. 345). This study was conducted in accordance with the Helsinki Declaration.

### Ti nanoparticle-induced calvarial osteolysis model

All animal experiments were approved by the Ethics Committee of the First Affiliated Hospital of Soochow University. Thirty 8–10 weeks C57BL/6 male mice were randomly divided into 3 groups: a sham operation + PBS treatment group (sham group), a Ti nanoparticle implantation + PBS treatment group (Ti group) and a Ti nanoparticle implantation + 1 mg/kg GKT137831 treatment group (Ti + GKT group). The size of Ti nanoparticles ranged from 24.51–233.58 nm (Additional file 1: Fig. S1A and B). A Ti nanoparticle-induced calvarial osteolysis model was established in the Ti-implantation groups. In brief, after anesthesia, the skin on the calvariae was shaved, disinfected and incised along the middle line, and then, the cranial periosteum was separated from the calvarium. Subsequently, 40 mg of Ti nanoparticles (40 μl) was evenly spread onto the surfaces of calvariae, and the incision was sutured. The mice in the sham group were subjected to the same surgical procedures without Ti nanoparticle suspension. Appropriate solutions were subperiosteal injected into the mice in different groups in the center of the calvariae once daily for 14 consecutive days. All mice were sacrificed 14 days after the operation, and calvariae were collected and fixed in 4% paraformaldehyde.

### Micro-CT analysis

After fixation, Ti nanoparticles on the surface of calvariae were removed to mitigate metal artifacts and then scanned using a Skyscan 1176 micro-CT device (Aartselaar, Belgium). NRecon software (Skyscan micro-CT, Aartselaar, Belgium) was employed to reconstruct two‐dimensional (2D) and three‐dimensional (3D) images, and Skyscan software was used to analyze bone parameters, including bone mineral density (BMD, mg/cm^3^), bone volume (BV, mm^3^), bone volume per tissue volume (BV/TV, %) and total porosity (%).

### Histological analysis and dihydroethidium (DHE) staining

Calvariae were decalcified in 10% EDTA, embedded in paraffin and prepared for histological sections. The sections were dewaxed with xylene, subjected to gradient hydration and subjected to hematoxylin and eosin (H&E, Beijing Leagene Biotechnology, China) and TRAP staining. Section images were acquired using an Axiovert 40C optical microscope (Zeiss, Germany). The number of TRAP-positive multinucleated osteoclasts in the selected regions of each group were quantified by ImageJ software (Bethesda, USA). DHE staining was performed according to the manufacturer’s protocol. In brief, the sections were dewaxed and hydrated, after which DHE staining was performed. Then, the nuclei were stained with DAPI, and images were captured using a fluorescence microscope and quantified using ImageJ software. RGB images were separated into single channels, and regions of interest (ROIs) were manually drawn and adjusted by threshold. Finally, the mean gray value of each channel was calculated. The fluorescence intensity (fold change) was the ratio of the fluorescence intensity in different groups.

### Statistics

The data are displayed as the mean ± standard deviation (SD). GraphPad Prism version 8.0 software was used for statistical analysis. The differences between two groups were compared using Student’s *t* test. One-way ANOVA was used to compare the differences among multiple groups. In the figures, error bars represent standard deviations. A p value < 0.05 was considered significant.

## Results

### NOX4 blockade inhibited RANKL-induced osteoclast differentiation

**Fig. 1:**
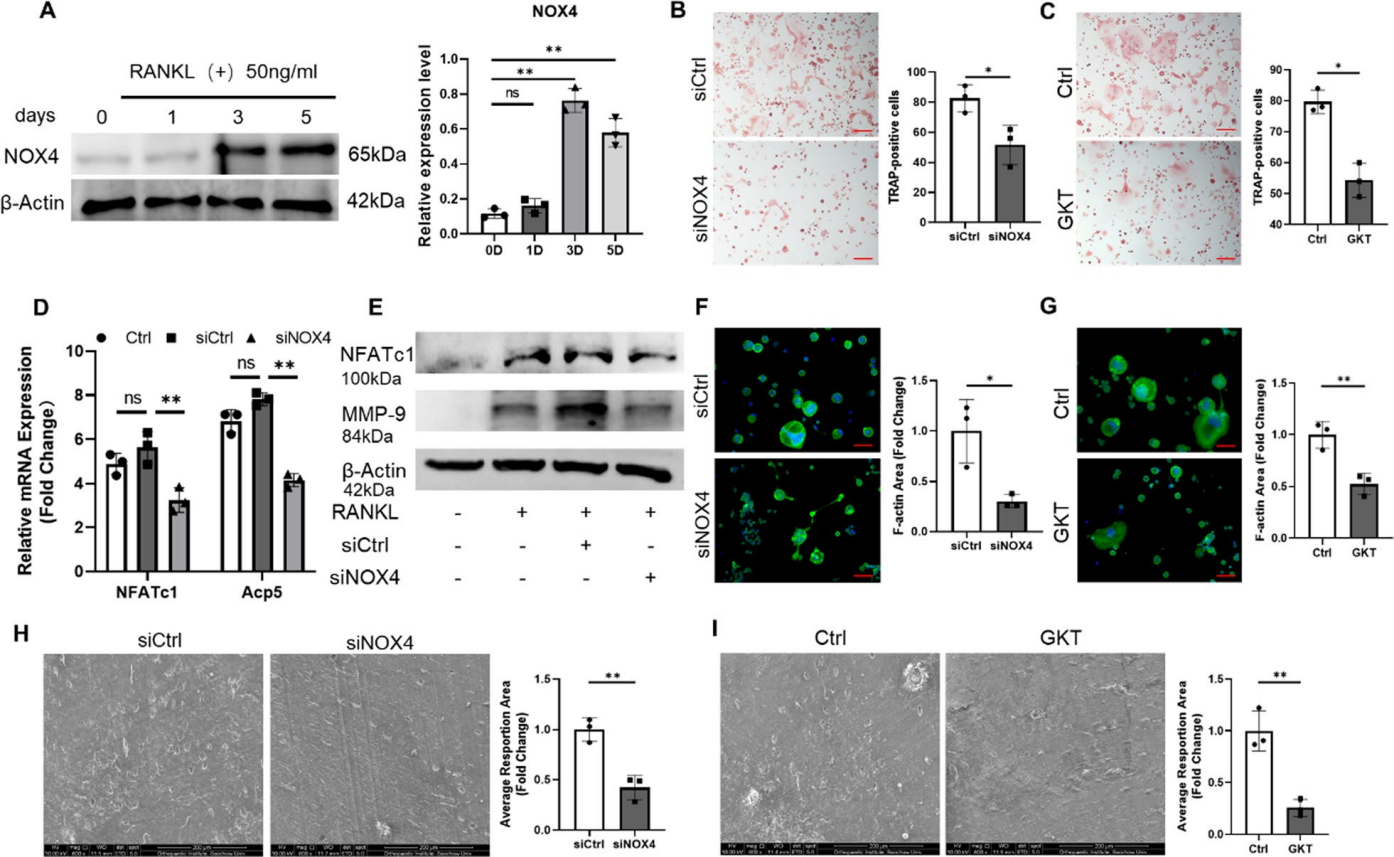
NOX4 blockade suppressed RANKL-induced osteoclastogenesis. **A** Protein levels of NOX4 during RANKL-induced osteoclastogenesis. **B** Representative TRAP staining images and quantification of TRAP-positive cells (≥ 3 nuclei) transfected with siNOX4 (**C**) and treated with GKT. Scale bars, 100 µm. **D** The expression of osteoclast-specific genes (**E**) and osteoclast-specific proteins. **F** Representative fluorescence images of F-actin rings after transfection with siNOX4 (**G**) and treatment with GKT. Scale bars, 50 µm. **H** Scanning electron microscopy (SEM) images of the bone resorption area after siRNA silencing (**I**) and after GKT treatment. Scale bars, 200 µm. ns: no significance, *p < 0.05, **p < 0.01

NOX4 expression during RANKL-induced osteoclastogenesis was measured to determine whether NOX4 is essential for osteoclast differentiation. We cultured RAW264.7 macrophages with RANKL (50 ng/ml), fused multinuclear cells were observed (Additional file 1: Fig. S2A), and the protein levels of the osteoclast-specific proteins NFATc1 and MMP-9 were found to increase during osteoclast differentiation (Additional file 1: Fig. S2B). NOX4 protein expression was also confirmed to gradually increase (Fig. 1A), which indicated that NOX4 expression is upregulated during RANKL-induced osteoclast differentiation.

To further determine the effects of NOX4 on RANKL-induced osteoclastogenesis, we transfected RAW264.7 cells with NOX4-specific small interfering RNA (siRNA) to silence NOX4. The silencing effect was demonstrated by western blotting (Additional file 1: Fig. S3). The transfected macrophages were then induced by 50 ng/ml RANKL, and TRAP staining revealed that NOX4 silencing strongly suppressed osteoclast differentiation (Fig. 1B). The specific NOX4 inhibitor GKT137831 (GKT) at 50 μM was also confirmed to reduce RANKL-induced osteoclastogenesis without detectable toxic effects (Fig. 1C and Additional file 1: Fig. S4). Then, the mRNA expression levels of the osteoclastic-specific markers NFATc1 and Acp5 (encoding the TRAP protein) were demonstrated to be inhibited following NOX4 silencing (Fig. 1D). In addition, we demonstrated that siNOX4 suppressed the expression levels of NFATc1 and MMP-9 via western blotting (Fig. 1E). Similar suppressive effects were found on RANKL-induced osteoclast differentiation with pharmacological inhibition of NOX4 (Additional file 1: Figure S5A and B).

Mature osteoclasts have bone resorptive abilities [21, 22], and thus, we investigated the effect of NOX4 blockade on osteoclastic function. The formation of an F-actin loop structure is required for bone resorption [23, 24], and a well-defined actin belt (green) was induced by RANKL stimulation. Both NOX4 silencing and pharmacological inhibition significantly decreased the actin ring and sealing zones (Fig. 1F and G). On the surface of the bovine bone slices, we observed that NOX4 blockade dramatically suppressed resorption, in contrast to the substantially increased resorption areas in the control group (Fig. 1H and I), indicating the destructive bone resorptive function of osteoclasts. Taken together, these data revealed that the expression of NOX4 is involved and necessary in RANKL-induced osteoclastogenesis and that NOX4 blockade inhibited RANKL-induced osteoclast differentiation and osteoclastic bone resorption.

### NOX4 blockade suppressed RANKL-induced ROS production

**Fig. 2:**
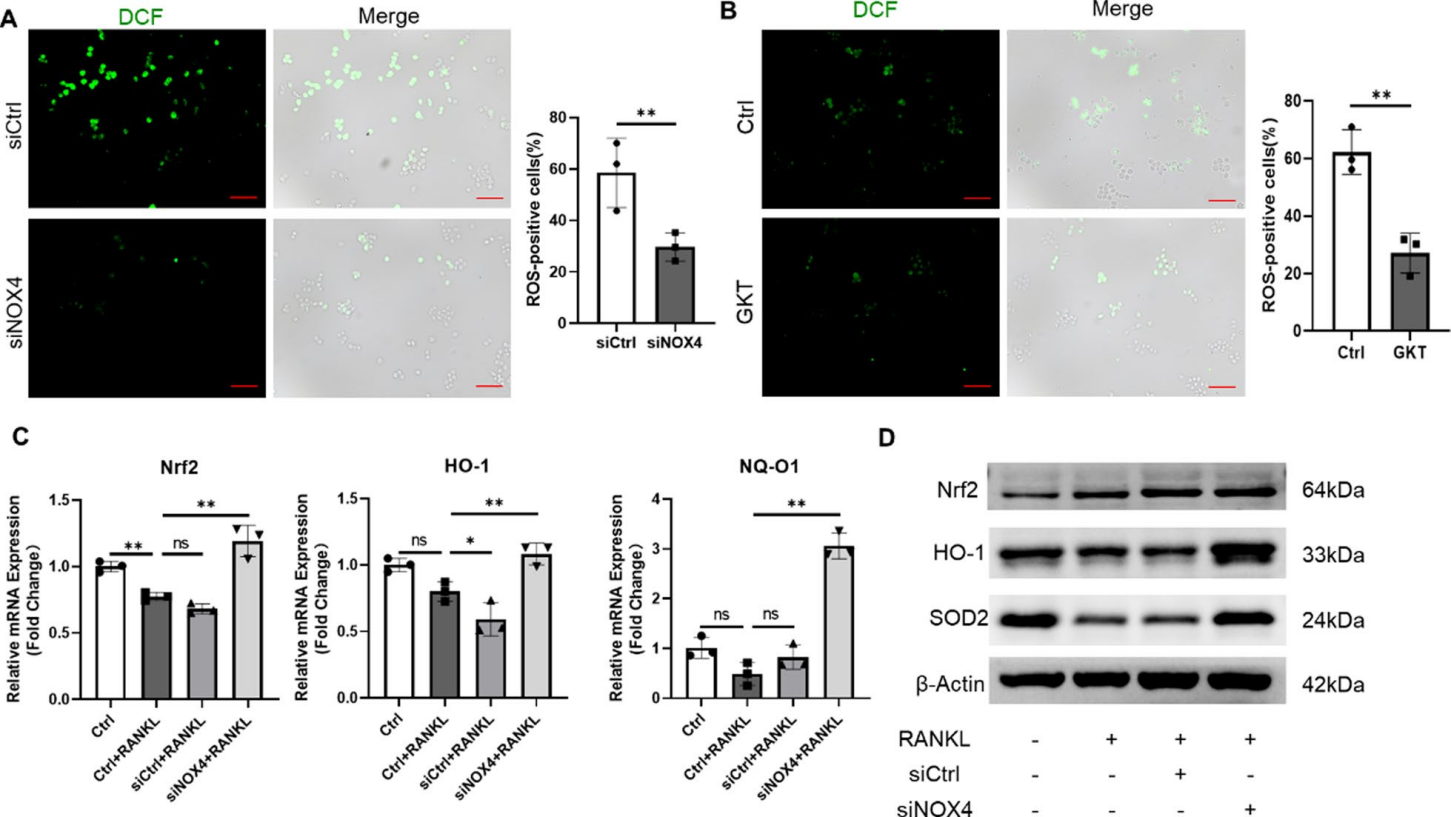
NOX4 blockade suppressed RANKL-induced ROS production. **A** Representative images of RANKL-induced ROS generation after siNOX4 silencing **B** and after GKT treatment. Scale bars, 50 µm. **C** The gene levels of Nrf2, HO-1, and NQ-O1 after siNOX4 silencing. **D** The protein levels of Nrf2, HO-1, and SOD2 after siNOX4 silencing. ns: no significance, *p < 0.05, **p < 0.01

Unbalanced ROS production promotes excessive osteoclastogenesis and robust bone resorption [10, 25]. To investigate whether NOX4 is involved in RANKL-induced ROS production and further promotes osteoclastic bone resorption, we treated RAW264.7 macrophages with RANKL and then stained them with DCFH-DA. The intensity of DCF fluorescence and the positive cell numbers were significantly increased after RANKL treatment. DCF fluorescence and positive cell numbers decreased in the RAW264.7 cells following transfection with NOX4 siRNA or treatment with GKT (Fig. 2A and B). There are various protective mechanisms by which cells scavenge ROS. Nrf2 plays an important role in oxidative stress, as the expression of several antioxidant enzymes is controlled by Nrf2 [26, 27], so we next investigated the expression of Nrf2 and its downstream factors. We demonstrated that Nrf2 expression was activated by NOX4 silencing during RANKL-induced osteoclastogenesis (Fig. 2C). Moreover, the expression levels of the Nrf2-downstream genes heme oxygenase-1 (HO-1) and NAD(P)H quinone oxidoreductase 1 (NQ-O1) were found to be upregulated (Fig. 2C). The western blot results revealed that NOX4 silencing also enhanced the protein levels of Nrf2 and HO-1 and the antioxidant stress protein superoxide dismutase 2 (SOD2) (Fig. 2D), indicating that Nrf2 and its downstream signals were involved in this process. Similarly, Nrf2 was activated by pharmacological inhibition of NOX4, thus leading to the upregulated expression of the HO-1 and NQ-O1 genes in different time periods (Additional file 1: Fig. S6A) and eventually increasing the protein expression of Nrf2, HO-1, and SOD2 for ROS scavenging (Additional file 1: Fig. S6B). Collectively, these data mechanistically revealed that NOX4 is involved in RANKL-induced ROS production, and NOX4 blockade suppressed RANKL-induced intracellular ROS via the suppression of ROS generation and the enhancement of ROS scavenging. Moreover, these results revealed that NOX4 may regulate RANKL-induced osteoclastogenesis via the overproduction of ROS and the expression of Nrf2.

### NOX4 regulated osteoclastogenic activity via ROS scavenging and Nrf2 activation

**Fig. 3:**
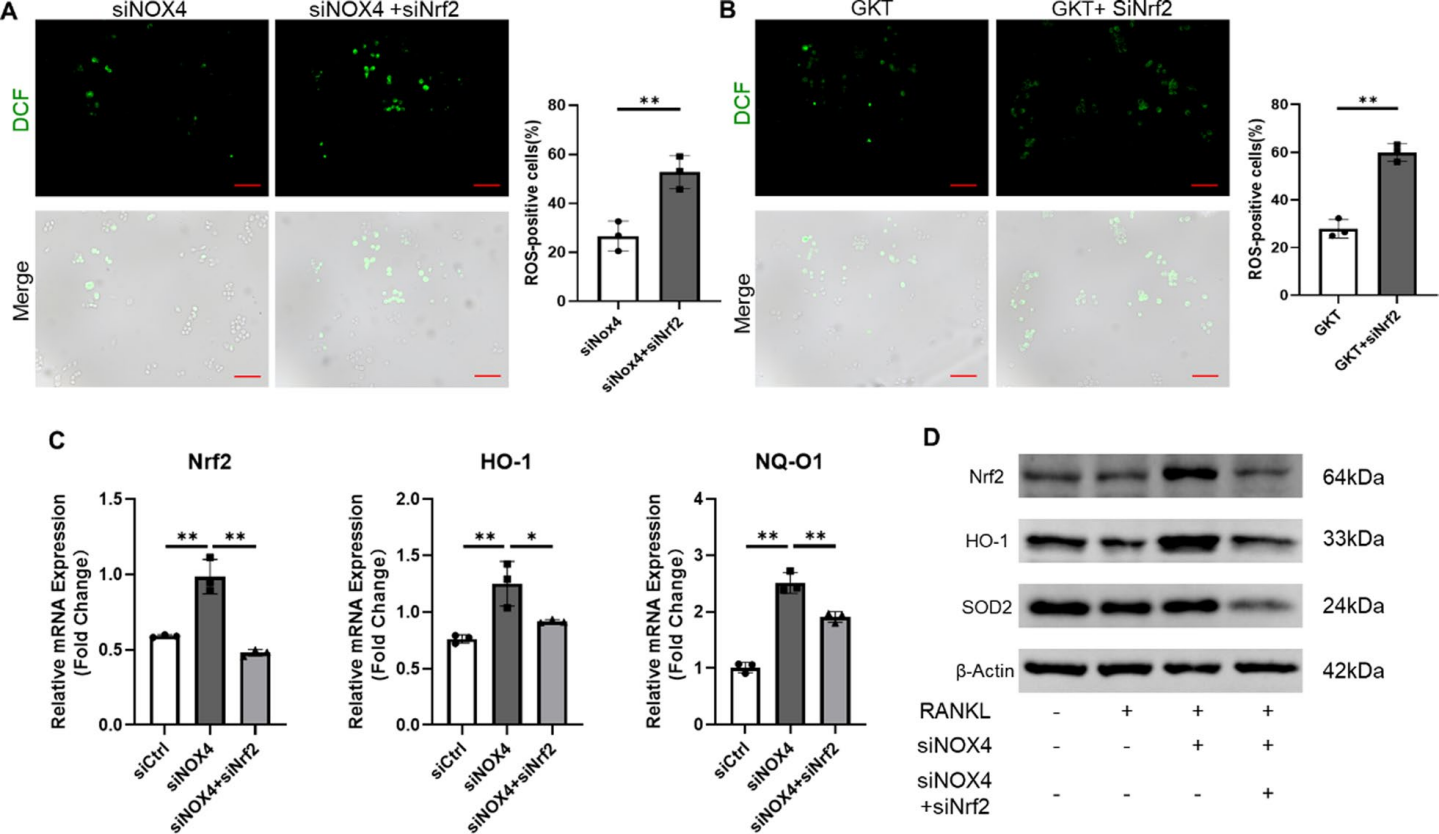
Silencing Nrf2 reversed the impaired RANKL-induced ROS production. **A** Representative images of RANKL-induced ROS generation in the RAW264.7 cells transfected with both siNrf2 and siNOX4 (**B**) or transfected with siNrf2 and pretreated with 50 μM GKT137831. Scale bars, 50 µm. **C** The gene levels of Nrf2, HO-1, and NQ-O1. **D** The protein levels of Nrf2, HO-1, and SOD2. *p < 0.05, **p < 0.01

We found that NOX4 blockade upregulated Nrf2 expression, which suggested the potential role of Nrf2 in these processes of RANKL-induced osteoclastogenesis. Nrf2 nuclear translocation is a prerequisite for the activation of downstream antioxidative target genes [27]. To investigate whether Nrf2 is involved in these processes, we silenced Nrf2 by Nrf2-specific siRNA (Additional file 1: Fig. S7). First, we found that the intensity of DCF fluorescence was significantly increased in the siNrf2-treated group compared with the siNOX4 or GKT group after the cells were stimulated with RANKL (Fig. 3A and B), indicating that downregulation of Nrf2 expression increased ROS production. Moreover, we detected Nrf2 and its downstream signals. The changes in the levels of the Nrf2, HO-1, and NQ-O1 genes (Fig. 3C and Additional file 1: Fig. S8A) and the Nrf2, HO-1, and SOD2 proteins (Fig. 3D and Additional file 1: Fig. S8B) were all reversed by Nrf2 silencing, which suggested impaired ROS scavenging. These data revealed that the benign effects of NOX4 blockade on ROS scavenging were reversed by Nrf2 silencing.

**Fig. 4:**
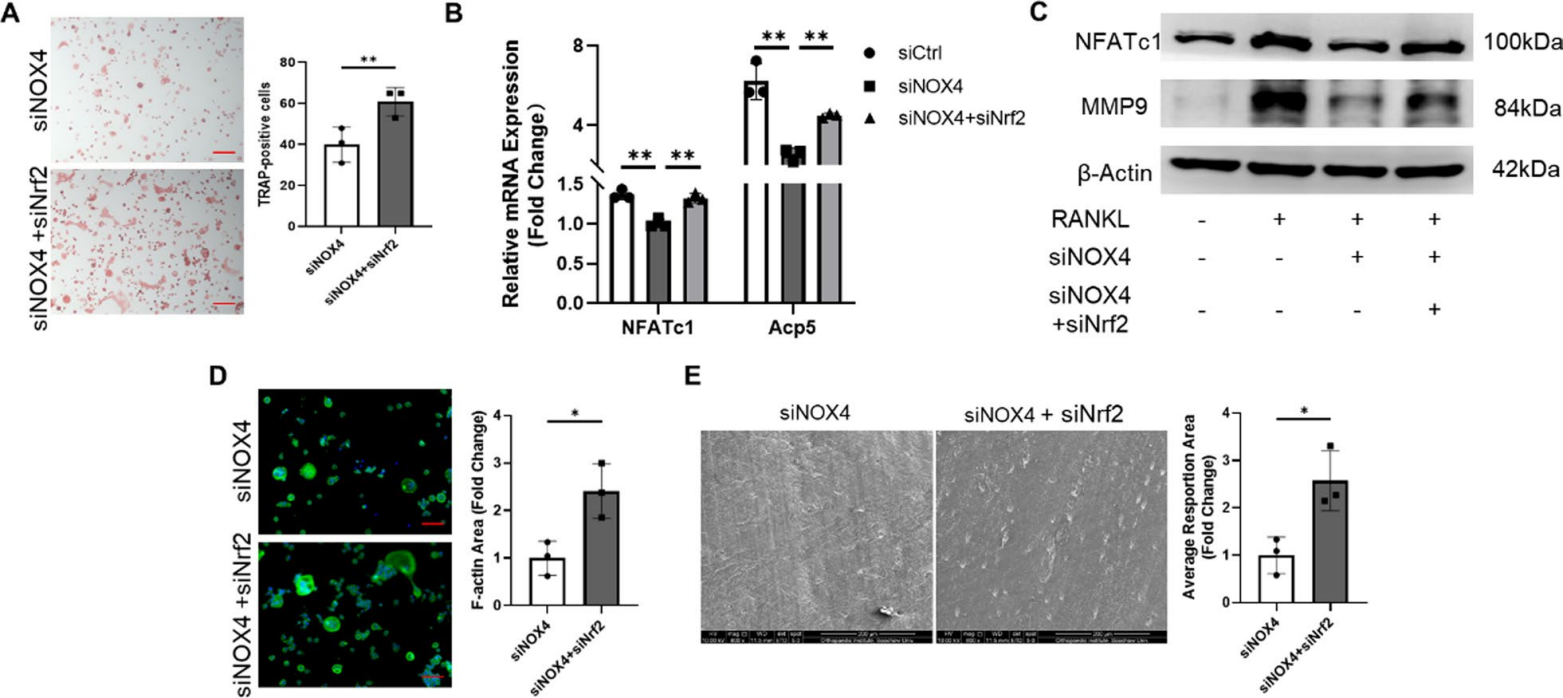
Silencing Nrf2 reversed the impaired RANKL-induced osteoclastogenesis. **A** Representative TRAP staining images and quantification of TRAP-positive cells transfected with both siNrf2 and siNOX4. Scale bars, 100 µm. **B** The expression of osteoclast-specific genes (**C**) and osteoclast-specific proteins. **D** Representative fluorescence images of F-actin rings. Scale bars, 50 µm. **E** Representative SEM images after cells were transfected with both siNrf2 and siNOX4. Scale bars, 200 µm. *p < 0.05, **p < 0.01

Since Nrf2 rescued the impaired RANKL-induced ROS production, we predicted that the changes in osteoclast differentiation and bone resorption would also be reversed. As expected, downregulation of Nrf2 expression attenuated the inhibitory effects of both siNOX4 and GKT on osteoclast differentiation, which was confirmed by TRAP staining (Fig. 4A and Additional file 1: Fig. S9A). In addition, we confirmed that the inhibitory effects of NOX4 silencing or pharmacological inhibition on the expression of osteoclastic genes were attenuated by Nrf2 silencing (Fig. 4B and Additional file 1: Fig. S9B). Interestingly, the downregulated levels of proteins related to osteoclast differentiation were found to be upregulated via the transfection of siNrf2 (Fig. 4C and Additional file 1: Fig. S9C). To verify whether Nrf2 silencing can reverse the inhibition of osteoclast functions, we used F-actin staining and bovine bone slices following the above methods. Silencing Nrf2 reversed the inhibition of osteoclastogenesis and resulted in the reappearance of mature osteoclasts (Fig. 4D and Additional file 1: Fig. S9D) as well as increased osteoclast bone resorption (Fig. 4E and Additional file 1: Fig. S9E), indicating a resurgence of osteoclastic resorption. Collectively, these results indicated that NOX4 blockade suppressed RANKL-induced osteoclastogenesis and ROS generation by enhancing the expression of Nrf2.

### Effect of NOX4 inhibition on PPO by preventing hyperactivity of osteoclasts in vivo

**Fig. 5:**
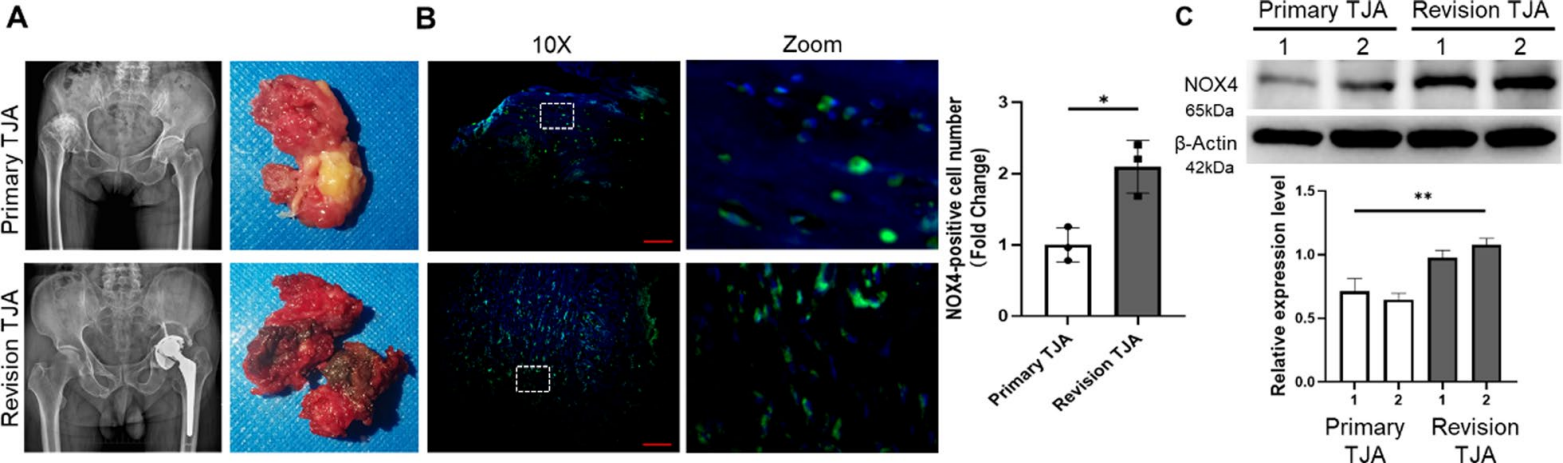
NOX4 is involved in wear particle-induced PPO. **A** Images of clinical tissues obtained from patients with primary TJA and revision TJA. **B** Representative images of immunofluorescence staining of NOX4-positive cells and quantification. Scale bars, 100 µm. **C** Protein levels of NOX4 in different samples. *p < 0.05, **p < 0.01

To investigate the effects of NOX4 in PPO, we collected periprosthetic tissues from patients with primary TJA and revision TJA (Fig. 5A). H&E staining demonstrated that a large number of wear particles appeared in the revision TJA tissues, and TRAP staining revealed increased TRAP-positive cells in the revision TJA tissues compared with the primary TJA tissues (Additional file 1: Fig. S10A and B). Immunofluorescence staining revealed that the expression of NOX4 in the revision TJA tissues was also significantly increased (Fig. 5B). Furthermore, the increased NOX4 protein levels in the revision TJA tissues were confirmed by western blotting (Fig. 5C). Collectively, these data suggested that NOX4 may be involved in wear particle-induced PPO.

**Fig. 6:**
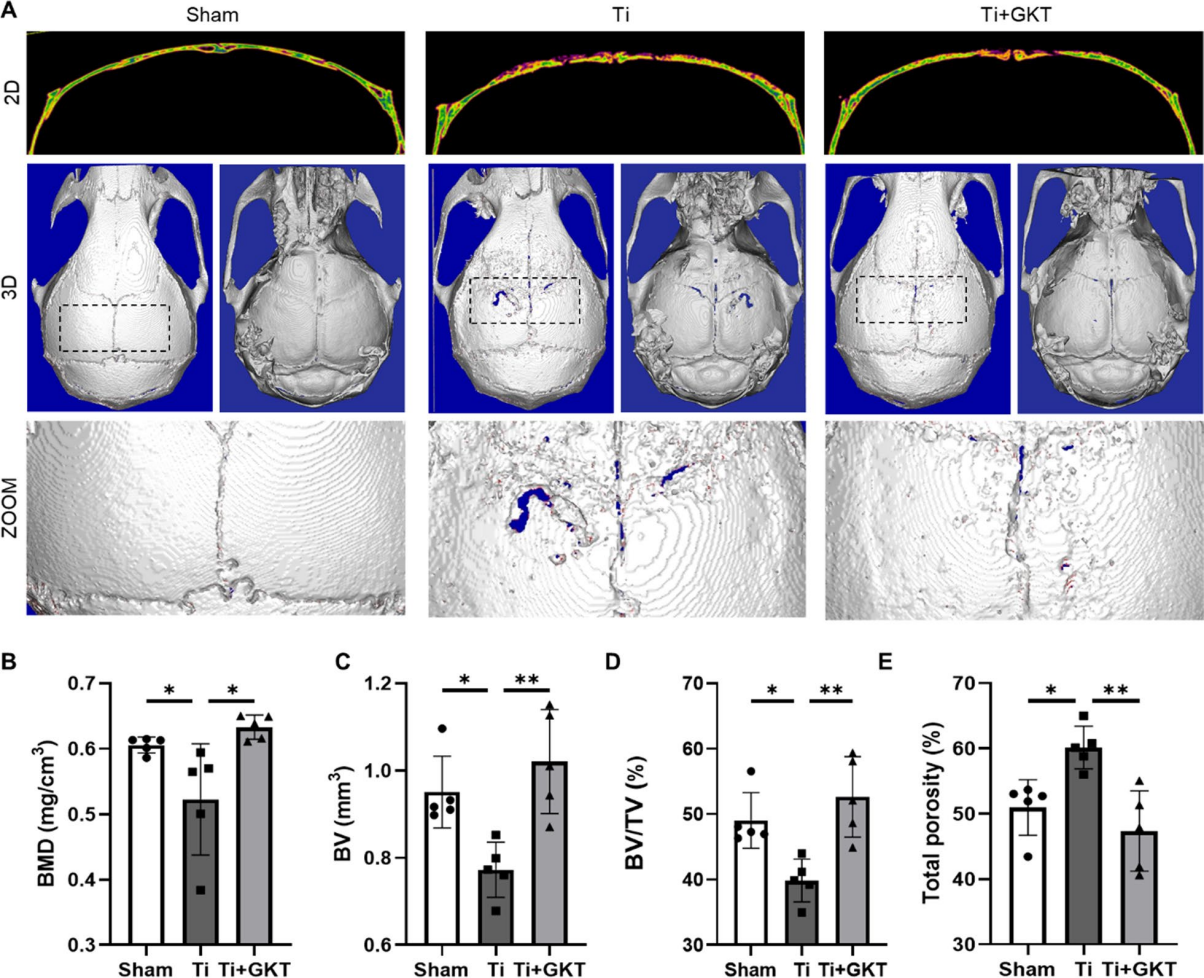
NOX4 inhibition alleviated Ti nanoparticle-induced bone destruction. **A** Representative 3D and 2D reconstruction images of micro-CT. **B** BMD (mg/cm^3^). **C** BV (mm^3^). **D** BV/TV (%). **E** Total porosity (%). *p < 0.05, **p < 0.01

We then investigated whether NOX4 blockade by GKT protected against nanoparticle-induced bone loss in an in vivo model of titanium nanoparticle-induced osteolysis (Additional file 1: Fig. S11). The 3D and 2D reconstruction images showed that calvariae in the Ti group were extensively eroded compared with those in the sham group. In contrast, these changes were obviously impaired by GKT therapy (Fig. 6A). Quantitative analysis of bone parameters revealed that BMD was dramatically reduced in the model group compared with the sham group (0.523 ± 0.085 mg/cm^3^ vs. 0.606 ± 0.012 mg/cm^3^, respectively), but GKT therapy inhibited the decrease in BMD (0.633 ± 0.018 mg/cm^3^) (Fig. 6B). In addition, the percentage of pores was increased and the BV and BV/TV values were markedly decreased in the Ti group compared with the sham group, while NOX4 inhibition impaired these parameters (Fig. 6C–E). H&E staining of the main organs showed no toxicity in the GKT treatment group (Additional file 1: Fig. S12).

**Fig. 7:**
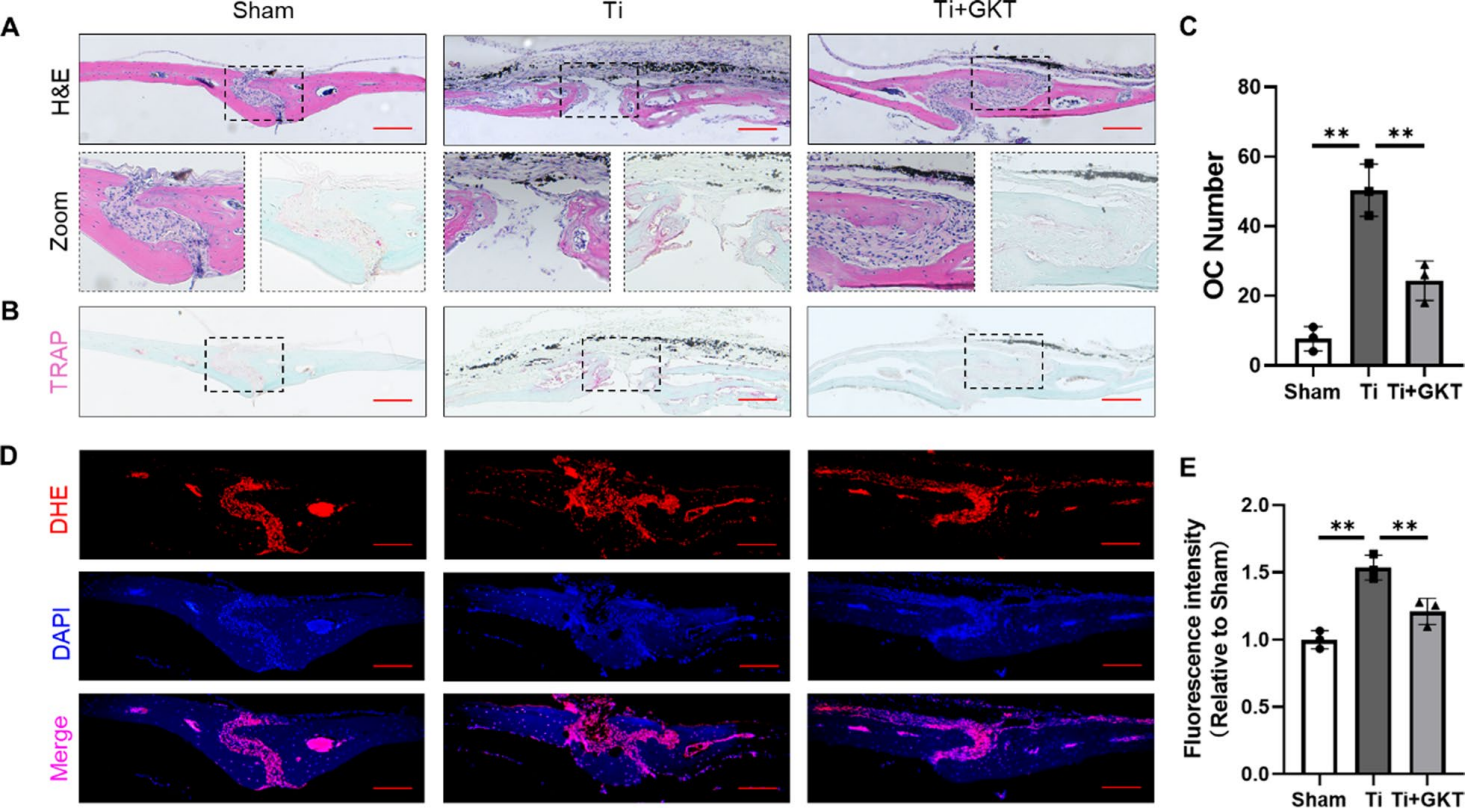
NOX4 inhibition moderated Ti nanoparticle-induced bone destruction in histological sections of the calvarial surface. **A** Representative images of H&E staining (**B**) and TRAP staining. Scale bars, 100 µm. **C** Quantification of the number of TRAP-positive osteoclasts. **D** Representative images of DHE staining. Scale bars, 50 µm. **E** Quantification of the DHE fluorescence intensity. **p < 0.01

Histological staining of calvarial sections was employed to detect the therapeutic effect of NOX4 inhibition on Ti particle-induced osteolysis. Consistent with the micro-CT results, the results of H&E staining revealed extensive erosion of the calvaria surface in the Ti group, which was markedly reduced by GKT (Fig. 7A). TRAP staining was also performed to detect the osteoprotective effect of GKT therapy. We found that GKT therapy dramatically decreased the number of osteoclasts compared with that in the model group (Fig. 7B and C). Then, the ROS probe DHE was used to assess ROS production on the bone surface in vivo. Similarly, ROS levels in bone tissue were obviously increased in the Ti group compared with the sham group, but GKT therapy significantly reversed ROS production (Fig. 7D and E). Taken together, these results demonstrated that NOX4 inhibition prevented Ti-induced osteolysis by decreasing ROS levels and osteoclastogenesis.

## Discussion

PPO is the most common complication of total joint replacement surgeries and an almost unavoidable issue that results in progressive bone destruction induced by wear particles [2, 12, 28]. Previous studies have suggested that hyperactive osteoclasts and increased oxidative stress level-induced bone resorption are predominant in the initiation of PPO, but the underlying mechanism remains incompletely understood, which increases the difficulty of treatment of PPO [12, 29, 30]. Traditional anti-resorption drugs had limited efficacy, as well as side effects [31–33]. Therefore, identification of novel therapeutic targets and efficacious drugs to prevent and treat PPO is urgently needed. Here, we demonstrated that NOX4-mediated ROS production and osteoclastogenesis are initiators in wear particle-induced PPO, while NOX4 blockade suppressed osteoclastogenesis and ROS production in vitro and Ti nanoparticle-induced osteolysis in vivo*,* identifying a potential therapeutic target to prevent and treat PPO.

ROS usually comprise superoxide anions, hydrogen peroxide and hydroxyl radicals [34], which are involved in the regulation of cell survival [35], proliferation [36], metabolism [37], apoptosis [38], differentiation [39], and migration [40] and play important roles in essential intracellular secondary messengers. However, under nanoparticle stimulation, the production of ROS was obviously enhanced in the peri-implant microenvironment, and excessive RANKL was secreted from osteoblasts [12, 41], promoting the progression of mature osteoclast formation and bone destruction on the surface of the peri-implant and eventually resulting in extensive osteolysis [6, 10, 42]. Thus, regulating excessive ROS production and osteoclastogenesis can be a strategy to treat PPO. Hu et al. revealed that suppressing ROS and RANKL production protected against osteolysis [43]. Sun et al. developed a few-layered Nb2C (FNC) as an antioxidant to scavenge ROS and inhibit osteoclastogenesis to attenuate osteolysis [12]. Xian et al. found that oroxylin A, a natural flavonoid isolated, prevented osteoclast-mediated osteolysis by suppressing RANKL-induced ROS and NFATc1 activation [13]. We demonstrated that the inhibition of NOX4, which contributes to the generation of ROS, prevented RANKL-induced osteoclastogenesis and oxidative stress to ameliorate wear particle-induced PPO. NOX4 contributes to the generation of ROS and directly produces H_2_O_2_, whereas other NOX proteins generate superoxide anions and require complex activation steps [11, 44]. More importantly, higher expression of NOX4 has been reported in mature osteoclasts than under basal conditions [45, 46]. This evidence indicates the dual roles of NOX4 in osteoclastogenesis and ROS generation in the pathogenesis of PPO. In our study, NOX4 blockade with siRNA or a specific inhibitor directly prevented RANKL-induced osteoclastogenesis and bone resorption; NOX4 inhibition decreased the intracellular ROS level and enhanced the expression of antioxidant enzymes, including Nrf2, HO-1, and SOD2, to scavenge ROS, which further suppressed osteoclast formation.

Nrf2 is a redox-sensitive transcription factor that regulates the expression of various antioxidant genes and proteins [26, 27]. Osteoclastogenesis was reported to be suppressed by Nrf2 overexpression [47, 48], whereas this process was induced by Nrf2 deficiency [49]. Scientists have confirmed that NOX4 maintains the level and activity of Nrf2 in the cardiovascular system and endothelial cells [44], but the relationship between Nrf2 and NOX4 in osteoclasts and the musculoskeletal system remains unknown. Although a direct association of endothelial cells and osteoclasts has not been found, both originate from monocytes. Hyeon et al. found that DPI, a strong NOX inhibitor, reversed the increased osteoclast formation and intracellular ROS levels in Nrf2-null cells [49]. In our study, we found that NOX4 inhibition attenuated osteoclastogenesis, enhanced ROS scavenging and upregulated Nrf2 expression, which suggested the potential role of Nrf2 in these processes. Therefore, we investigated whether Nrf2 and its downstream signaling components participate in the regulation of NOX4. The results are consistent with our hypothesis, which showed that downregulating Nrf2 expression reversed the impaired RANKL-induced ROS production and osteoclastogenesis.

**Fig. 8:**
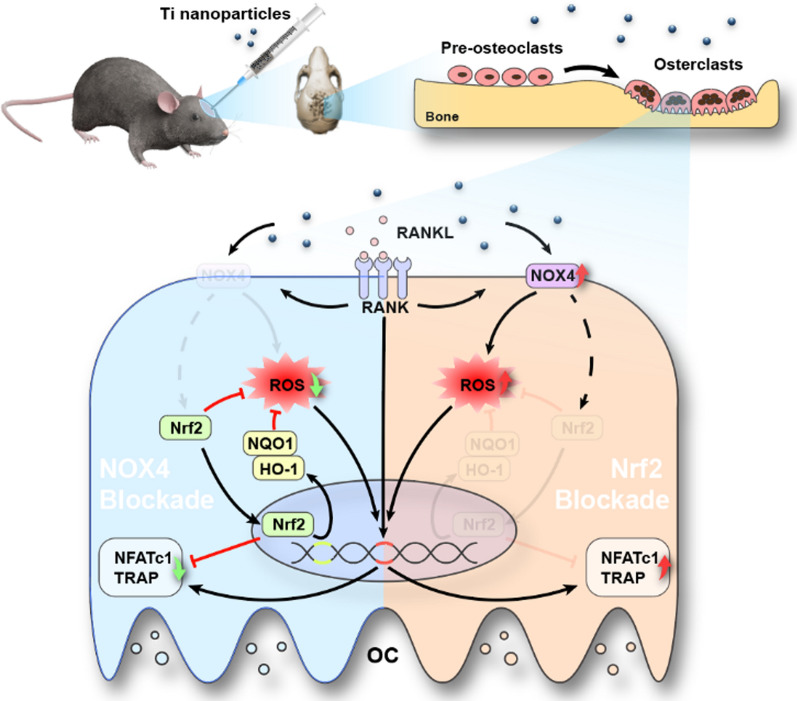
Schematic illustration of the relative mechanism of the inhibitory effect of NOX4 blockade on osteoclastogenesis

Interestingly, in clinical tissues from patients who underwent revision TJA, we found that NOX4 was expressed at significantly higher levels, suggesting a critical role for NOX4 in PPO. Similar modulation of NOX4 was identified in previous studies showing that NOX4 expression was upregulated in pulmonary diseases, cardiovascular diseases, kidney injury and other conditions [50–52]. In the current study, we further investigated whether NOX4 inhibition could protect against Ti nanoparticle-induced bone loss. The micro-CT reconstruction and H&E staining images indicated that calvariae from the Ti group were extensively eroded compared with those from the sham group; in contrast, this change was dramatically impaired after GKT treatment. We also demonstrated that GKT treatment not only suppressed osteoclastogenesis in Ti-induced osteolysis but also scavenged overproduced ROS on the bone surface. Micro-CT quantification and histological and immunofluorescence analyses showed interesting results: the data between the Ti + GKT group and the sham group did not show a linear relationship, indicating there may be other contributing mechanisms. In addition to activating osteoclastogenesis, wear debris suppresses osteogenesis by inhibiting osteoblast function and promoting osteoblast apoptosis via the production of ROS [53–55]. Moreover, previous studies have revealed the role of NOX4 in ROS production and osteogenesis [56, 57]. Based on these findings, we predicted that NOX4 blockade rescued Ti nanoparticle-induced osteogenic inhibition by direct or indirect protective effects. GKT137831 inhibits the inflammatory response of macrophages and alleviating the inflammatory environment may be another potential mechanism because NOX4 has been shown to promote NLR family pyrin domain containing 3 (NLRP3) activation in macrophages [58]. In the future, it will be essential to explore the potential role of NOX4 in ameliorating inflammation and promoting osteogenesis. Collectively, we found that NOX4 inhibition prevented Ti nanoparticle-induced osteolysis by reducing the production of ROS and osteoclastogenesis both in vivo and in vitro (Fig. 8).

Our study has several limitations. Ti nanoparticles were used to induce calvarial osteolysis because of their stability and adhesion. Ti nanoparticles and polyethylene debris can comparably trigger PPO, but polyethylene debris easily floats. However, in the clinic, the majority of wear particles are polyethylene debris [59–62]. Thus, the effects of NOX4 blockade on polyethylene debris-induced osteolysis should be further explored. Moreover, we silenced NOX4 with siRNA, which was identified as an effective way to evaluate the effects of NOX4 blockade on osteoclastogenesis and ROS production and explored the possible involved mechanisms. However, this method is not easy to apply in clinical trials under the existing conditions. In the future, we would do some surface modifications together with NOX4 siRNA or inhibitor coating for local delivery to the bone sites slowly and orderly. In the meanwhile, we will attempt to apply the clinical trial of GKT137831 for the treatment of PPO. Although our calvarial model is classic and widely adopted and can effectively simulate PPO, the calvarial model differs from long bone and lacks weight-bearing capacity and fluid pressure. The mechanism of bone formation/resorption in calvarial bone is also different from that in the long limbs. The Ti rod with nanoparticles implanted into the larger animal femur could be a more appropriate model to mimic the clinical features of PPO in our further studies.

## Conclusion

Our findings demonstrate the function of NOX4 in the process of wear particle-mediated PPO. NOX4 blockade suppresses bone resorption by preventing hyperactivity of osteoclasts. The enhanced ROS scavenging and the activation of Nrf2 and its downstream signaling pathway could be the underlying mechanism. In addition, NOX4 blockade suppresses titanium nanoparticle-induced bone destruction through anti-osteoclastic and antioxidant activities. Collectively, these findings suggested NOX4 inhibition may be an attractive therapeutic approach for preventing PPO.

## Supplementary Information

**Additional file 1: Table S1.** Primers used in RT-PCR. **Figure S1. **(**A**) Representative scanning electron microscopy (SEM) image of Ti nanoparticles. Scale bar, 500 nm. (**B**) Frequency distribution of Ti nanoparticle size. **Figure S2. **RANKL-induced osteoclast differentiation. (**A**) Representative TRAP staining images during RANKL-induced osteoclastogenesis. Scale bars, 100 µm. (**B**) The protein levels of the osteoclast-specific proteins NFATc1 and MMP-9. **Figure S3.** The silencing effect of NOX4 siRNA confirmed by western blot and quantification analysis. ns: no significance, **p < 0.01. **Figure S4. **The cytotoxicity of GKT137831 on RAW264.7 macrophages was detected by CCK-8 kit. ns: no significance. **Figure S5.** GKT suppressed RANKL-induced osteoclastogenesis. (**A**) The gene levels of NFATc1 and Acp5. (**B**) The protein levels of NFATc1 and MMP-9. **p < 0.01. **Figure S6.** GKT upregulated the expression of Nrf2 and its downstream signal. (**A**) The genes of Nrf2, HO-1, and NQ-O1 in different time periods. (**B**) The proteins of Nrf2, HO-1, and SOD2. ns: no significance, *p < 0.05, **p < 0.01. **Figure S7.** The silencing effect of Nrf2 siRNA confirmed by western blot and quantification analysis. *p < 0.05, **p < 0.01. **Figure S8.** Silence Nrf2 reversed the upregulated effects of GKT on Nrf2 and its downstream signal. (**A**) The genes of Nrf2, HO-1, and NQ-O1. (**B**) The proteins of Nrf2, HO-1, and SOD2. ns: no significance, *p < 0.05, **p < 0.01. **Figure S9.** Silence Nrf2 reversed the inhibitory effects of GKT on osteoclast differentiation. (**A**) Representative TRAP staining images and quantification when transfected with siNrf2 and pretreated with 50 μM GKT. Scale bars, 100 µm. (**B**)The gene expression of NFATc1 and Acp5. (**C**) The protein expression of NFATc1 and MMP-9. (**D**) Representative F-actin ring images (**E**) and representative SEM images after cells were transfected with siNrf2 and pretreated with 50 μM GKT137831. Scale bars, 50 µm and 200 µm respectively. *p < 0.05, **p < 0.01. **Figure S10.** Histological staining for the sections of clinical tissues. Representative images of (**A**) H&E staining and (**B**) TRAP staining from primary TJA and revision TJA. Scale bars, 100 µm. **Figure S11.** Schematic diagram of in vivo model. **Figure S12.** H&E staining of the organ tissue sections (Heart, lung, and kidney). Scale bars, 100 µm.

## Data Availability

The datasets generated and/or analyzed during the current study are not publicly available but are available from the corresponding author on reasonable request.

## Abbreviations

TJR: Total joint replacement
PPO: Periprosthetic osteolysis
NOX4: Nicotinamide adenine dinucleotide phosphate oxidase 4
Nrf2: Nuclear factor-erythroid 2-related factor 2
RANKL: Receptor activator of nuclear factor-κ B ligand
TRAP: Tartrate-resistant acid phosphatase
MMP-9: Matrix metalloprotein-9
ROS: Reactive oxygen species
NFATc1: Nuclear factor of activated T cells cytoplasmic 1
CCK8: Cell Counting Kit-8
HO-1: Heme oxygenase-1
NQ-O1: NAD(P)H quinone oxidoreductase 1
SOD2: Superoxide dismutase 2
DCFH-DA: 2,7-Dichlorodihydrofluorescein diacetate
BMD: Bone mineral density
DHE: Dihydroethidium
