# Supplementary material for: NOX4 blockade suppresses titanium nanoparticle-induced bone destruction via activation of the Nrf2 signaling pathway
Source: J Nanobiotechnology. 2022 May 23;20:241. doi: 10.1186/s12951-022-01413-w (PMC9125939; doi:10.1186/s12951-022-01413-w)
Supplement: Supplementary file 1 — Additional file 1: Table S1. Primers used in RT-PCR. Figure S1. (A) Representative scanning electron microscopy (SEM) image of Ti nanoparticles. Scale bar, 500 nm. (B) Frequency distribution of Ti nanoparticle size. Figure S2. RANKL-induced osteoclast differentiation. (A) Representative TRAP staining images during RANKL-induced osteoclastogenesis. Scale bars, 100 µm. (B) The protein levels of the osteoclast-specific proteins NFATc1 and MMP-9. Figure S3. The silencing effect of NOX4 siRNA confirmed by western blot and quantification analysis. ns: no significance, **p < 0.01. Figure S4. The cytotoxicity of GKT137831 on RAW264.7 macrophages was detected by CCK-8 kit. ns: no significance. Figure S5. GKT suppressed RANKL-induced osteoclastogenesis. (A) The gene levels of NFATc1 and Acp5. (B) The protein levels of NFATc1 and MMP-9. **p < 0.01. Figure S6. GKT upregulated the expression of Nrf2 and its downstream signal. (A) The genes of Nrf2, HO-1, and NQ-O1 in different time periods. (B) The proteins of Nrf2, HO-1, and SOD2. ns: no significance, *p < 0.05, **p < 0.01. Figure S7. The silencing effect of Nrf2 siRNA confirmed by western blot and quantification analysis. *p < 0.05, **p < 0.01. Figure S8. Silence Nrf2 reversed the upregulated effects of GKT on Nrf2 and its downstream signal. (A) The genes of Nrf2, HO-1, and NQ-O1. (B) The proteins of Nrf2, HO-1, and SOD2. ns: no significance, *p < 0.05, **p < 0.01. Figure S9. Silence Nrf2 reversed the inhibitory effects of GKT on osteoclast differentiation. (A) Representative TRAP staining images and quantification when transfected with siNrf2 and pretreated with 50 μM GKT. Scale bars, 100 µm. (B)The gene expression of NFATc1 and Acp5. (C) The protein expression of NFATc1 and MMP-9. (D) Representative F-actin ring images (E) and representative SEM images after cells were transfected with siNrf2 and pretreated with 50 μM GKT137831. Scale bars, 50 µm and 200 µm respectively. *p < 0.05, **p < 0.01. Figure S10. Histological staini [file 12951_2022_1413_MOESM1_ESM.docx]

**NOX4 blockade** **suppresse****s** **titanium nanoparticle-induced bone destruction via activation of the Nrf2 signaling pathway**

*Wei Wang ^1,#^,* *Xiaolong Liang ^1,#^, Xin Liu* *^1,#^, Jiaxiang Bai ^1^, Wei Zhang ^1^,* *Wenming Li ^1^, Tianhao Wang* *^1^, Meng Li ^1,2^, Zerui Wu* *^1,3^, Liang Chen ^1^, Huilin Yang ^1^, Ye Gu ^4,^*, Yunxia Tao ^1,^*, Jun Zhou ^1,^*, Huaiyu Wang ^5^, Dechun Geng ^1,4,^**

*^1^* Department of Orthopedics, The First Affiliated Hospital of Soochow University, Suzhou, Jiangsu 215006, China.

*^2^* Department of Orthopedic Surgery, The First Affiliated Hospital of University of Science and Technology of China, Hefei, Anhui 230001, China.

*^3^* Department of Orthopedic Surgery, The Affiliated Hospital of Xuzhou Medical University, Xuzhou, Jiangsu, 221002, China.

*^4.^* Department of Orthopedics, Changshu Hospital Affiliated to Soochow University, First People’s Hospital of Changshu City, Changshu, China.

*^5^* Center for Human Tissues and Organs Degeneration, Shenzhen Institute of Advanced Technology, Chinese Academy of Sciences, Shenzhen 518055, China.

# These authors contributed equally to this work

* Corresponding authors should be addressed to:

szgengdc@suda.edu.cn (Dechun Geng), zhou.jun.roy@hotmail.com (Jun Zhou),

taoyx8493@163.com (Yunxia Tao), edwinguye@126.com (Ye Gu).

Supplementary Materials

**Table S1.** Primers used in RT-PCR.

| Gene | Primer Sequence (F) | Primer Sequence (R) |
| --- | --- | --- |
| NFATc1 | GAGAATCGAGATCACCTCCTAC | TTGCAGCTAGGAAGTACGTCTT |
| Acp5 | TGTGGCCATCTTTATGCT | GTCATTTCTTTGGGGCTT |
| Nrf2 | CAGCCATGACTGATTTAAGCAG | CAGCTGCTTGTTTTCGGTATTA |
| HO-1 | AGGTACACATCCAAGCCGAGA | CATCACCAGCTTAAAGCCTTCT |
| NQ-O1 | AGGATGGGAGGTACTCGAATC | AGGCGTCCTTCCTTATATGCTA |
| GAPDH | GGTTGTCTCCTGCGACTTCA | TGGTCCAGGGTTTCTTACTCC |


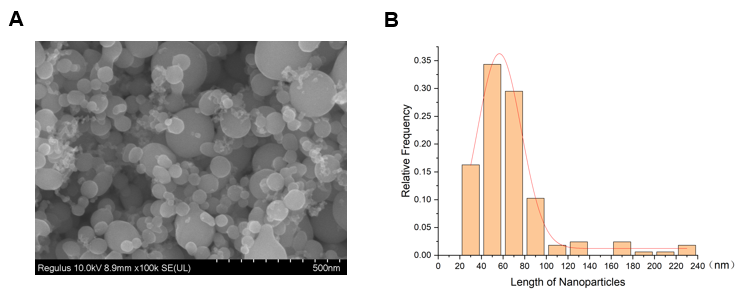


**Figure S1.** (**A**) Representative scanning electron microscopy (SEM) image of Ti nanoparticles. Scale bar, 500 nm. (**B**) Frequency distribution of Ti nanoparticle size.


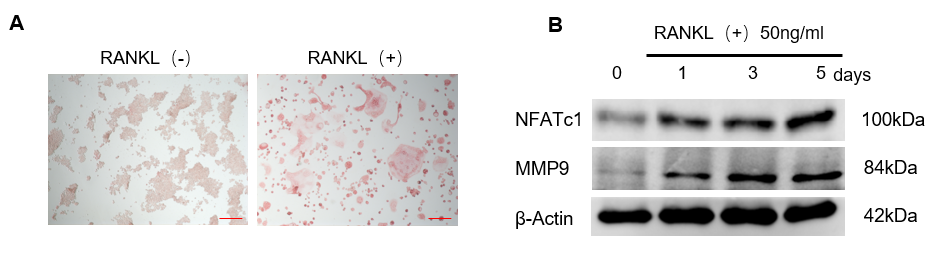


**Figure S2.** RANKL-induced osteoclast differentiation. (**A**) Representative TRAP staining images during RANKL-induced osteoclastogenesis. Scale bars, 100 µm. (**B**) The protein levels of the osteoclast-specific proteins NFATc1 and MMP-9.


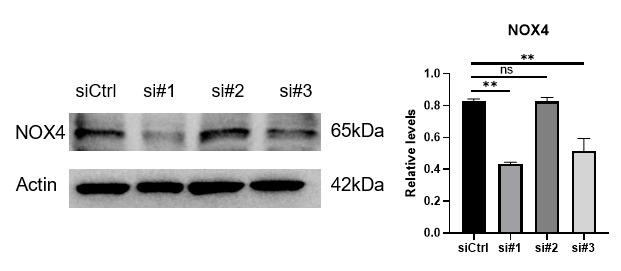


**Figure S3.** The silencing effect of NOX4 siRNA confirmed by western blot and quantification analysis. ns: no significance, **p < 0.01.


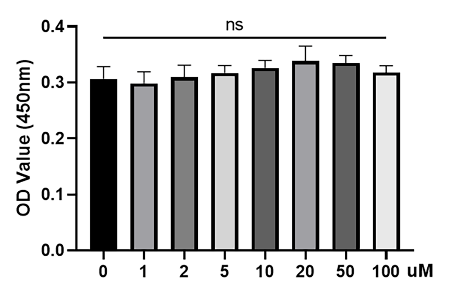


**Figure S4.** The cytotoxicity of GKT137831 on RAW264.7 macrophages was detected by CCK-8 kit. ns: no significance.


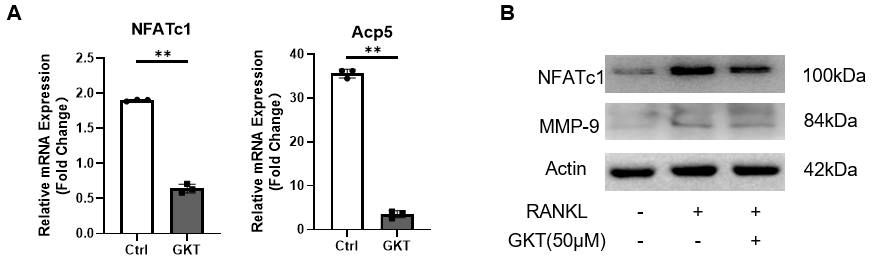


**Figure S5.** GKT suppressed RANKL-induced osteoclastogenesis. (**A**) The gene levels of NFATc1 and Acp5. (**B**) The protein levels of NFATc1 and MMP-9. **p < 0.01.


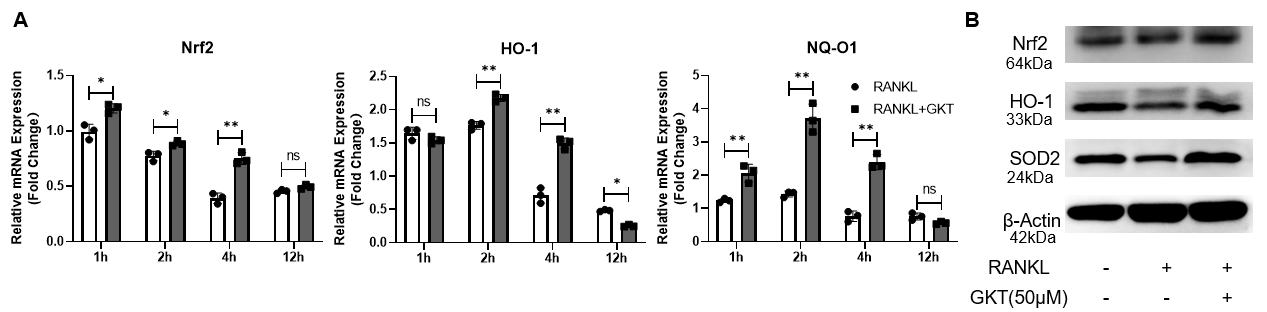


**Figure S6.** GKT upregulated the expression of Nrf2 and its downstream signal. (**A**) The genes of Nrf2, HO-1 and NQ-O1 in different time periods. (**B**) The proteins of Nrf2, HO-1 and SOD2. ns: no significance, *p < 0.05, **p < 0.01.


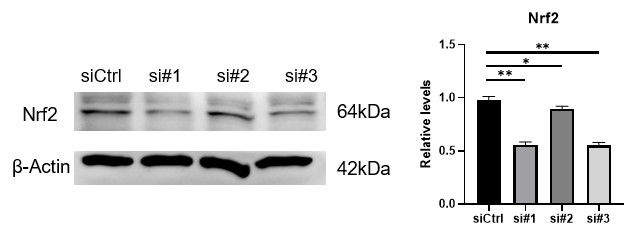


**Figure S7.** The silencing effect of Nrf2 siRNA confirmed by western blot and quantification analysis. *p < 0.05, **p < 0.01.


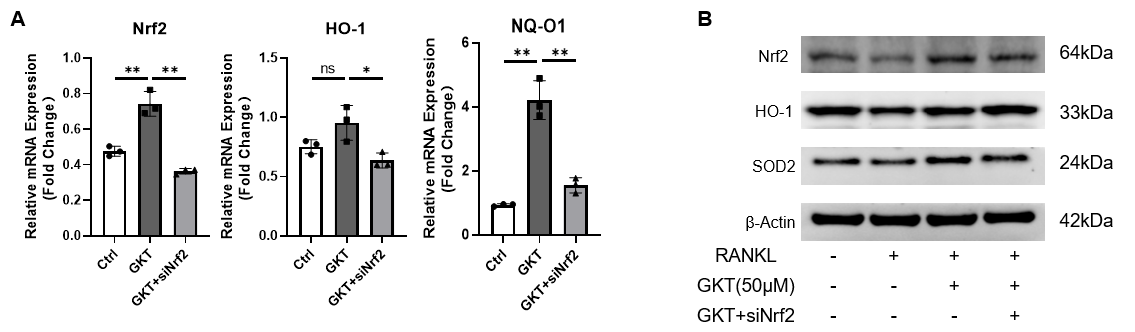


**Figure S8.** Silence Nrf2 reversed the upregulated t effects of GKT on Nrf2 and its downstream signal. (**A**) The genes of Nrf2, HO-1 and NQ-O1. (**B**) The proteins of Nrf2, HO-1 and SOD2. ns: no significance, *p < 0.05, **p < 0.01.


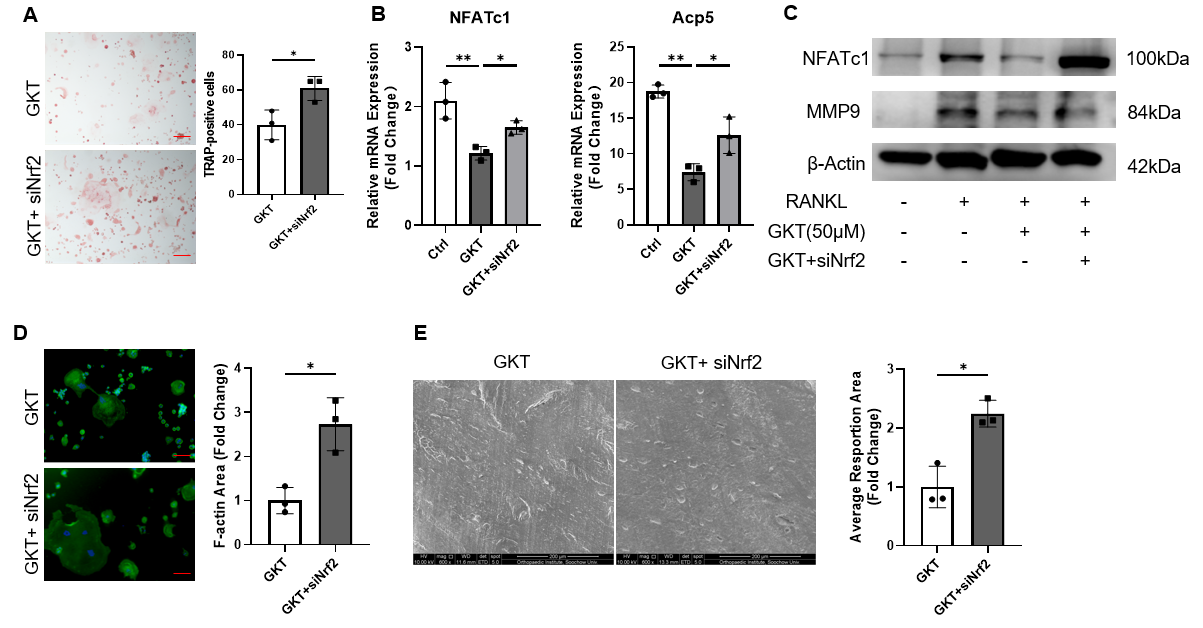


**Figure S9.** Silence Nrf2 reversed the inhibitory effects of GKT on osteoclast differentiation. (**A**) Representative TRAP staining images and quantification when transfected with siNrf2 and pretreated with 50 μM GKT. Scale bars, 100 µm. (**B**)The gene expression of NFATc1 and Acp5. (**C**) The protein expression of NFATc1 and MMP-9. (**D**) Representative F-actin ring images (**E**) and representative SEM images after cells were transfected with siNrf2 and pretreated with 50 μM GKT137831. Scale bars, 50 µm and 200 µm respectively. *p < 0.05, **p < 0.01.


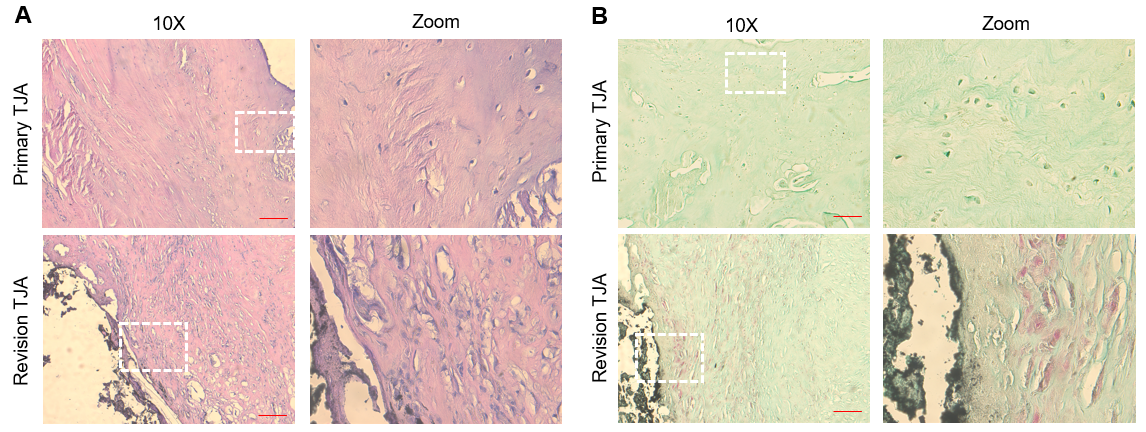


**Figure S10.** Histological staining for the sections of clinical tissues. Representative images of (**A**) H&E staining and (**B**) TRAP staining from primary TJA and revision TJA. Scale bars, 100 µm.


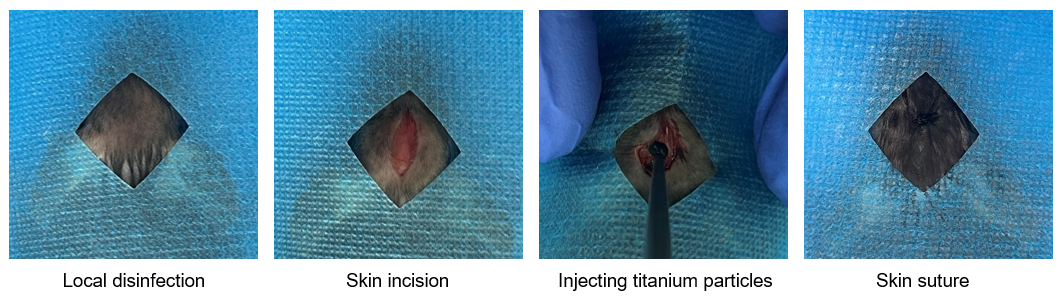


**Figure S11.** Schematic diagram of *in vivo* model.


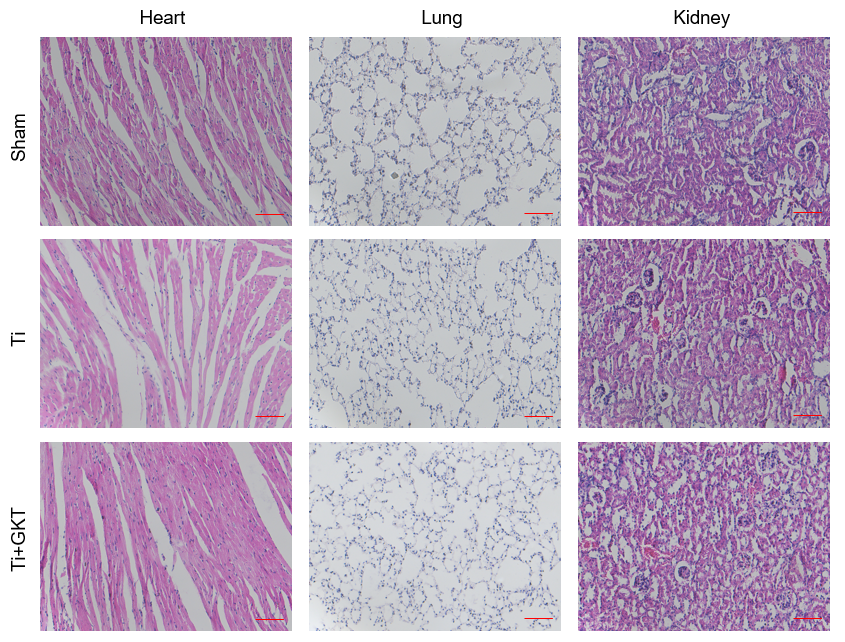


**Figure S12.** H&E staining of the organ tissue sections (Heart, lung, and kidney). Scale bars, 100 µm.
